# The reading-attention relationship: Variations in working memory network activity during single word decoding in children with and without dyslexia

**DOI:** 10.1016/j.neuropsychologia.2024.108821

**Published:** 2024-02-08

**Authors:** Niki Sinha, C. Nikki Arrington, Jeffrey G. Malins, Kenneth R. Pugh, Jan C. Frijters, Robin Morris

**Affiliations:** a Department of Child and Youth Studies, Brock University, St. Catharines, ON, L2S 3A1, Canada; b Department of Psychology, Georgia State University, Atlanta, GA, 30303, United States; c GSU/GT Center for Advanced Brain Imaging, Georgia State University, Atlanta, GA, 30318, United States; d Center for Translational Research in Neuroimaging and Data Science, Georgia State University, Atlanta, GA, 30303, United States; e Haskins Laboratories, New Haven, CT, 06511, United States; f Department of Linguistics, Yale University, New Haven, CT, 06520, United States; g Department of Psychological Sciences, University of Connecticut, Storrs, CT, 06269, United States

**Keywords:** Reading, Working memory, Developmental dyslexia, fMRI, Decoding, Children

## Abstract

This study utilized a neuroimaging task to assess working memory (WM) network recruitment during single word reading. Associations between WM and reading comprehension skills are well documented. Several converging models suggest WM may also contribute to foundational reading skills, but few studies have assessed this contribution directly. Two groups of children (77 developmental dyslexia (DD), 22 controls) completed a functional magnetic resonance imaging (fMRI) task to identify activation of *a priori* defined regions of the WM network. fMRI trials consisted of familiar word, pseudoword, and false font stimuli within a 1-back oddball task to assess how activation in the WM network differs in response to stimuli that can respectively be processed using word recognition, phonological decoding, or non-word strategies. Results showed children with DD recruited WM regions bilaterally in response to all stimulus types, whereas control children recruited left-lateralized WM regions during the pseudoword condition only. Group-level comparisons revealed activation differences in the defined WM network regions for false font and familiar word, but not pseudoword conditions. This effect was driven by increased activity in participants with DD in right hemisphere frontal, parietal, and motor regions despite poorer task performance. Findings suggest the WM network may contribute to inefficient decoding and word recognition strategies in children with DD.

## 1. Introduction

Reading is understood to occur from the coordinated effort of multiple cognitive processes. Individual foundational reading skills and their interdependent influence on development forms the basis of many reading models. The most widely known is the simple view of reading, which suggests reading comprehension is dependent on the individual development of decoding and language comprehension skills (Gough and Tunmer, 1986). Recent literature expands the simple view into more comprehensive models which consider additional underlying cognitive skills that act as a foundation to reading, and the interactions between them (Duke and Cartwright, 2021; Hulme and Snowling, 2016; Reynolds, 2000). Executive functions encompass a range of cognitive processes that contribute to the effective execution of foundational reading skills, and may play an additional independent role through self regulatory processes (Cirino et al., 2019; Follmer, 2018; Jacobson et al., 2017; Locascio et al., 2010). Working memory (WM) is a capacity-limited system which defines executive functions involved in the temporary maintenance and manipulation of new information to complete complex tasks (Goldstein and Naglieri, 2014; McCabe et al., 2010). WM has been well established as a strong predictor for reading comprehension in children as young as nine (Jacobson et al., 2017; Locascio et al., 2010; Miller et al., 2014), but the exact mechanisms through which it contributes to reading development are not yet understood. There are two routes through which WM is suggested to interact with reading development – by contributing directly to foundational reading skills such as phonological decoding (Arrington et al., 2014; Chen et al., 2016), or by coordinating different reading processes to facilitate successful reading comprehension (Aboud et al., 2018; Fischbach et al., 2014; Peng et al., 2018).

Phonological decoding is a skill that helps readers identify new or unfamiliar words and is a predictor of later reading success (Facoetti et al., 2010). Effective decoding requires readers to hold letters in memory, pair them with the sounds they make, and order them correctly to ‘sound out’ a word. This is a complex process for new readers that requires support from the WM system to maintain and organize visual and phonetic information (Demoulin and Kolinsky, 2016; Smirni et al., 2020; Snowling and Hulme, 2012). In languages with opaque spelling systems, such as English, readers must also learn to apply rules in which letters correspond with multiple sounds (e.g. **c**at and **c**yan), or groups of letters take on entirely new ones (e.g. m**ight**, f**olk**). The added complexity of opaque sound-spelling rules highlights the need for an efficient WM system to maintain verbal information while accessing contextual information that must be explicitly learned (Arrington et al., 2014; Dehaene, 2009; Ehri, 2005).

Given explicit reading instruction and practice (Harm and Seidenberg, 2004; Siegelman et al., 2020) phonetic decoding becomes automatic, and less demand is placed on verbal WM to pair letter symbols and sounds (Dehaene, 2009). Reduced reliance on WM support during single word reading results in greater reading fluency and allows the capacity-limited WM system to contribute towards more complex processes involved in reading comprehension, such as inference making and accessing prior knowledge (Lavie, 2010; Morey and Cowan, 2005). WM is a known correlate of reading comprehension regardless of reading level (Follmer, 2018), but has been shown to contribute to phonetic decoding and word recognition individually as well (Jacobson et al., 2017; Lyon et al., 2003). In the typical reader, WM supports the development of foundational reading skills, *and* the coordination of cognitive skills necessary for effective reading comprehension (Kendeou et al., 2009).

The question then remains, how does WM contribute to the relative development of struggling readers? WM has been a significant predictor for poor reading intervention response in early childhood (Al Otaiba and Fuchs, 2006; Fletcher et al., 2011). Children with developmental dyslexia (DD) often show deficits in verbal WM when compared to typically developing peers, with a strong correlation found between verbal WM performance and single word decoding in this population (Knoop-van Campen et al., 2018; Locascio et al., 2010; Vaughn et al., 2020). Poor decoders struggle more with reading comprehension and fluency tasks compared to fast decoders or slow but accurate decoders (Facoetti et al., 2010; Hamilton et al., 2016). This is thought to be due to increased WM demands as reading material becomes more complex. WM is a time and capacity limited system and cannot hold information in indefinite quantities without being actively maintained (Baddeley and Hitch, 1974; Thomason et al., 2009). If WM remains necessary to support decoding in struggling readers, then attentional resources will strain as reading demands increase (Follmer, 2018; Knoop-van Campen et al., 2018; Reynolds, 2000).

However, the relationship between WM and reading development is not a strictly linear one. Although WM remains a significant predictor for reading comprehension and academic success across all age groups (Daucourt et al., 2018; Follmer, 2018; Gerst et al., 2017), it does not appear to adequately predict for discrete improvements in reading ability across intervention (Miciak et al., 2019). Additionally, reading interventions which include a focus on WM training have found improvements in WM skill have little or no effect on reading gains (Church et al., 2019; Dion et al., 2011; Gerst et al., 2017). This is somewhat surprising, given deficits in verbal WM is a consistent finding among children with DD (Beneventi et al., 2010; D’Mello and Gabrieli, 2018; Hulme and Snowling, 2016). It remains inconclusive whether children with DD show a general deficit in WM capacity (Facoetti et al., 2010; Fischbach et al., 2014), or one that is secondary to implicit deficits in phonological awareness (Church et al., 2019; Roe et al., 2018). While studies have shown children with DD perform more poorly on tasks that assess verbal WM capacity compared to their typically developing peers (Beneventi et al., 2010; Fischbach et al., 2014), this relationship does not always persist when phonological awareness is included as a mediator (Knoop-van Campen et al., 2018). Recently, evidence from neuroimaging studies have suggested WM may act as a compensatory mechanism in children with DD (Aboud et al., 2018; Nugiel et al., 2019). Compensation may be effective in early childhood when reading demands are relatively low, but become less practical as reading material increases in complexity and cognitive demand.

Given the capacity limits of WM during reading acquisition, allocation of attentional resources towards early reading skills may differ greatly between strong and poor readers. Contributions of WM to early reading development have been previously difficult to define in cognitive research due to the overlap in behavioural measures used to define single word reading and verbal WM (Cirino et al., 2017; Miciak et al., 2019; Stuebing et al., 2002). Functional neuroimaging provides a promising alternative as reading and WM-related activity can be identified through individual neural correlates, allowing researchers to track their relative neural contributions to a task (Aboud et al., 2018; Arrington et al., 2019). Functional magnetic resonance imaging (fMRI) localizer tasks, such as the one described in Malins et al. (2016) have shown effective in stratifying regions of the reading network based on their sensitivity to different types of word information within a single task. A following study by Arrington et al. (2019) further showed a similar task could be used to isolate regions maximally sensitive to reading from those sensitive to executive control. By looking at functional activity during single word reading using the same paradigm, we may begin to better understand key differences in the way WM contributes to foundational reading skills between strong and poor readers.

### 1.1. Defining anatomical networks for reading and working memory

Skilled reading occurs over a specialized reading network which shows a predominant pattern of left lateralization in strong readers (D’Mello and Gabrieli, 2018). The reading network is often described through its three circuits: 1) the temporo-parietal/dorsal circuit which engages in effortful, rule-based decoding, 2) the occipito-temporal/ventral circuit which engages in fast visual recognition of words, and 3) the anterior circuit which supports dorsal regions with the effortful decoding of new and/or challenging words (Barquero et al., 2014; Pugh et al., 2001). The anterior circuit is mainly described through the inferior frontal gyrus (IFG), which uses subvocal rehearsal to scaffold phonological decoding (Boisgueheneuc et al., 2006; Hoeft et al., 2011). Typical reading development begins with high dorsal and anterior activity that decreases over time. As readers gain experience, they begin to rely more heavily on fast word recognition, maintained through high connectivity between dorsal and ventral regions (Morken et al., 2017; Wise Younger et al., 2017).

Working memory activates a bilateral fronto-parietal network which partially overlaps with the defined reading network. This overlap provides additional methodological challenges in separating measurements of reading skills from those of verbal WM. Nevertheless, converging analyses have stratified neural correlates of WM that are specific to the processing of verbal and non-verbal stimuli, as well as those that are characteristic of a core central executive (Emch et al., 2019; Rottschy et al., 2012). In adults, it is primarily the anterior circuit of the reading network which overlaps with verbal processing regions of the working memory network. Regions thought to be specific to verbal WM tasks include the left IFG as well as the right CrusI of the cerebellum. Both of these regions have been associated with subvocal rehearsal of verbal information across a number of studies (Emch et al., 2019; Mariën et al., 2014). The anterior circuit plays an active role in supporting dorsal network activity via verbal WM, as such, overlap of the left IFG between the reading network and working memory network is expected and enduring (Pugh et al., 2001).

In children, overlap between these two networks is more variable. The middle frontal gyrus (MFG), and rostral prefrontal cortex (rPFC) are typically associated with general WM activity attributed to the central executive in mature networks (Rottschy et al., 2012), but become associated with verbal WM activity in children who are still learning to read (Paulesu et al., 2014; Weiss-Croft and Baldeweg, 2015). Children with DD show unique differences in functional activity between the reading network and regions associated with non-verbal WM. The most replicated finding relates to increases to volume and activity of the right IFG (Barquero et al., 2014; Chyl et al., 2021; Phan et al., 2021), as well as increases in connectivity between the right IFG and left lateralized reading network (Hoeft et al., 2011). Right IFG hyperactivity can be found in pre-reading children who go on to develop DD, indicating it does not occur because of reading difficulties (Chyl et al., 2021). However, increased right IFG activity is linked with stronger response to reading interventions (Aboud et al., 2018; Hoeft et al., 2011). Increased activity in frontal WM network regions may play a similar scaffolding role to the left IFG which comprises the reading network’s anterior circuit. The WM network – and particularly the right IFG in children with DD – may therefore act as a compensatory mechanism in young and/or struggling readers.

### 1.2. Current study

This study used an fMRI localizer task to isolate activity across the WM network during single word decoding. We aimed to identify reading-related differences in WM network activity between children with and children without DD. The task used different types of stimuli to induce specific strategies of word reading: pseudowords, which must be phonologically decoded; familiar words, which can be identified through orthographic recognition or phonological decoding; and false font “words” which acted as a non-verbal control (Malins et al., 2016). In addition, we examined brain-behaviour correlations between WM network activity and individual differences in subskills of verbal WM. Through this assessment we may begin to characterize the specific processes through which WM contributes to reading skill. If WM supports single word reading through an alternative mechanism in DD children, we expect to see recruitment of different regions of the WM network under different stimuli.

WM recruitment during single word reading is most evident during phonological decoding (Facoetti et al., 2010; Reynolds, 2000). As such, it was expected that pseudowords would elicit activity in the WM network for both DD and control groups, but that differences between groups would be maximal in response to familiar word stimuli. In addition, DD participants were expected to show poorer task performance in pseudoword and familiar word trials. Increased WM activity in response to pseudoword and familiar word stimuli may indicate WM directly supports decoding skill in DD participants. However, increased WM activity in response to all stimulus types may instead indicate WM plays a more generalized role in single word reading.

## 2. Methods

### 2.1. Participants

Participants were recruited from elementary schools in the greater Atlanta area in Georgia. Recruitment was part of a larger longitudinal study on reading intervention approved by the Joint Georgia State University/Georgia Tech Center for Advanced Brain Imaging Institutional Review Board. DD participants were recommended to the study after being identified as struggling readers by their teachers (Arrington et al., 2019). Parents and their children provided informed consent/assent prior to participation. All participants were primary English speakers in the 3rd or 4th grade at the time of recruitment. The average age of participants was 9.3 years (min = 7.8, max = 11.3). To control for intellectual disabilities, all participants had a standard score ≥80 for verbal and performance intelligence using the Wechsler Abbreviated Scale of Intelligence – Second Edition (WASI-II). Children with chronic absenteeism, uncorrected vision, or serious neurological, emotional, or psychiatric conditions or MRI contraindication were excluded from the study as indicated by screening questionnaires completed by parents. Ninety-nine participants (56 male, 43 female) were included in the current study as having complete relevant behavioural and imaging data prior to intervention in the larger longitudinal study.

#### 2.1.1. Behavioural measures

Composite scores from four reading measures were used to assess participant reading skill. All four reading composites assess foundational reading skills. The Woodcock Johnson III (WJ3) basic composite standard scores measures word reading accuracy through letter-word identification and word attack subtests, while the WJ3 broad composite standard scores use letter word identification, passage comprehension, and reading fluency subtests to evaluate reading comprehension. The Test of Word Reading Efficiency – Second Edition (TOWRE-2) measures reading fluency through phonemic decoding and sight word reading subtests, finally the Scholastic Reading Inventory – Second Edition (SRI2) includes passage comprehension and word recognition subtests to assess reading comprehension. Participants were classified as DD (n = 77) if they scored 1 standard deviation below age-related norms on any of the four standardized reading composites. Participants who scored within or above the normal range were classified as TD (n = 22). Groups did not differ in distribution of age or gender. In addition, participants were assessed for differences in attention via the NEPSY-II auditory attention subtest, and in non-verbal intelligence via the WASI-II matrix reasoning subtest (Table 1).

Subtests of the California Verbal Learning Test – Children’s Version (CVLT-C) were used as a behavioural measure of verbal working memory. The CVLT-C pairs word list learning and recall performance to assess memory and executive functions through encoding, memory storage, and memory rehearsal (Cutting et al., 2003; Fine and Delis, 2018). The test consists of five learning trials through which participants memorize a list of words through rehearsal as well as intermittent inclusion of an interference list which shares semantic commonality. Both target and interference list are presented as shopping lists and include 15 words under three semantic categories each (Fine and Delis, 2018). Learning trials test immediate recall of target list and short-term storage after intrusion. Following the learning period, recall of the target list is measured after a long delay period of 20 min and yes/no recognition testing of target list words is administered. Recall is tested via free recall which relies entirely on participant storage and retrieval, and cued recall in which participants are given semantic prompts (Fine and Delis, 2018). WM has been identified as a significant contributor to CVLT encoding and memory scores in both adult and child populations (Cutting et al., 2003; Lundervold et al., 2019).

The current study examined three CVLT-C measures: percent recall consistency across learning trials, long delay cued recall of target list words, and correct recognition of target words following learning and interference trials. Exploratory factor analysis in previous research has shown the CVLT-C has a three-factor internal structure for elementary school children with DD. These factors were named executive functioning, verbal memory, and inaccurate recall (Riggall, 2020). Percent recall consistency was the primary contributor to the executive functioning factor in Riggall’s (2020) model, while long delay cued recall and correct recognition hits were both significant contributors to the verbal memory factor. Through inclusion of these three measures this study sought to identify brain-behaviour correlations specific to verbal memory and executive functioning individually.

### 2.2. Magnetic resonance imaging

#### 2.2.1. Image acquisition

Images were acquired using a 3T Siemens scanner located at the GSU/GaTech Center for Advanced Brain Imaging in Atlanta, Georgia. The site scanner was upgraded from a Trio (12-channel head coil) to a PRIMSA-Fit (20-channel) during the final year of data collection (*n* = 13). T2*-weighted images were collected in an axial-oblique orientation parallel to the intercommissural line (32 slices; 4 mm slice thickness; no gap) using single-shot echo planar imaging (matrix size = 64 × 64; voxel size 3.438 × 3.438 × 4 mm; FoV = 220 mm; TR = 2000ms; TE = 30ms; flip angle = 80°). The first six volumes of each run were discarded to allow for the stabilization of the magnetic field. Time between trial onset was jittered between 4 and 13 s across all trials in the study. Each participant completed two to four runs of the functional task, with four runs being the optimal outcome. There were eleven cases where only three runs, and eight cases where only two runs were collected, due to issues of timing or participant discomfort. Each run was 316 s in duration (158 vol) for a maximum of 21 min of imaging time. Anatomical scans were collected either between or following functional runs in the same orientation (MPRAGE; matrix size = 256 × 256; voxel size = 1 × 1 × 1 mm; FoV = 256 mm; TR = 2530ms; TE = 2.77ms; flip angle = 7°). Imaging data were excluded if they did not meet quality control thresholds, most often due to excessive movement (Malins et al., 2016).

To account for differences between data collected before and after the scanner upgrade, data were harmonized using ComBat (Johnson et al., 2007). ComBat assumes the imaging feature measurements can be modeled as a linear combination of biological variables, with the scanner effects as an error term that includes a multiplicative scanner-specific scaling factor. It has been shown to effectively reduce inter-scanner variation in MRI data while effectively preserving biological associations (Fortin et al., 2017, 2018).

#### 2.2.2. Reading/attention task

Participants completed a single word reading task with inclusion of an oddball trial to simultaneously measure attention, known as the “fast” localizer reading-attention task (FastLoc-R/A). The FastLoc-R/A task was first used in Arrington et al. (2019) and is in turn a modified version of the “fast” localizer task developed by Malins et al. (2016). The task was designed to isolate regions of the reading network maximally sensitive to different types of word information (Malins et al., 2016). With added attentional component the FastLoc-R/A can further localise regions of the working memory network sensitive to familiar words, non-familiar words, and non-verbal stimuli (Arrington et al., 2019). Within the task, participants were presented with four runs of 48 trials. In each trial, four visual items were presented one after the other in rapid fashion replicating a passive reading experience. Participants silently read the items and were instructed to press a button with their right thumb if the fourth item in the trial was a repetition of the third. No response was required during standard trials where all four items differed. Trials with repeating items occurred in 33% of trials across all stimulus conditions and were defined as oddball trials, with an incorporated 1-back effect. The 1-back effect is distinct from an oddball (or 0-back) effect as participants are required to recall previous items in order to respond correctly to the oddball cue (Owen et al., 2005). Previous research has shown n-back tasks are effective in measuring functional WM activity in children and adults (Beneventi et al., 2010; Vogan et al., 2016; Yaple and Arsalidou, 2018). Previous research has also shown FastLoc-R/A task engages brain regions associated with attentional control independently of reading trials (Arrington et al., 2019).

**Fig. 1.**
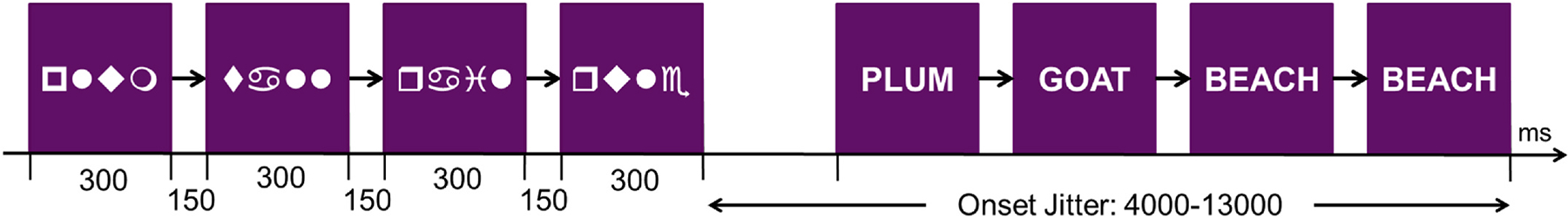
Trial sequence of a standard trial in the false font condition followed by an oddball trial in the familiar word condition (Arrington et al., 2019).

Visual stimuli occurred in tetrads, with each item appearing alone on screen for 300ms followed by an ISI of 150ms, and an onset jitter between trials of 4000–13000ms (Fig. 1; Arrington et al., 2019). Three stimulus conditions were included in the current study: familiar word, pseudoword, and false font (Wingdings), out of six visual conditions present in the overall study. Included items were balanced for length, frequency, bigram frequency, and deemed familiar for children (Arrington et al., 2019; further details provided in Malins et al., 2016). Across four runs, there were a maximum of 24 trials for each stimulus condition per participant.

Participants were familiarized with the task prior to the scanning session by completing a shortened version of the task in a mock scanner. Task performance was assessed via accuracy of response to trial types, such that correct responses were defined on oddball trials as a button press occurring between 250 and 2250ms post-stimulus onset, and on standard trials as a withheld response between 250 and 2250ms. Expected reaction times were adjusted for younger participants (see Arrington et al., 2019). Mean accuracy was calculated for each participant by comparing the number of correct responses over the total number of trials completed. Performance accuracy was measured for each stimulus type individually as well as for the task as a whole.

### 2.3. Statistical analysis

Preprocessing of single-subject and group level data was largely consistent with Arrington et al. (2019), with the exception that data were transformed to the Haskins Pediatric template to reduce non-linear deformation distance in the subject group (Molfese et al., 2021). Following preprocessing, *a priori* regions of interest (ROIs) were chosen within the WM network using converging data from a coordinate-based meta-analysis by Rottschy et al. (2012). The meta-analysis identified main effect WM via peaks of convergent activation across 189 fMRI experiments on healthy controls, and further stratified region specific sensitivity to verbal or non-verbal stimuli, as well as task-set or load dependent effects (Rottschy et al., 2012). ROIs were directly selected from coordinates of peak activation related to main effect WM provided in Rottschy et al. (2012), with additional consideration to regions listed as sensitive to verbal or non-verbal stimuli as well as task-set effects (contrasting WM trials with control trials). Data were analyzed using AFNI (Cox, 1996). Peak coordinates were transformed from MNI to Haskins Pediatric space (*3dNWarpXYZ*) and used to create spherical ROIs with a centered 6 mm radius (*3dUndump*). Beta values were extracted for all participants using *3dROIstats* such that each value represented the voxel average across the ROI. Values were extracted for correct standard and oddball trials only across familiar word, pseudoword, and false font conditions. To reduce confounding activity due to the reading demands associated with the task, WM activity was isolated by subtracting standard trials from oddball trials. After subtraction, beta values significantly above or below 0 represented a meaningful difference in BOLD activation within the WM network during correctly identified oddball trials compared to standard reading trials.

ROIs chosen to reflect main effect WM include the middle frontal gyrus (MFG), rostral prefrontal cortex (rPFC), right inferior frontal gyrus (IFG), and intraparietal sulcus (IPS), as well as the superior parietal lobule (SPL) which has shown specific association with recognition tasks such as the oddball (Rottschy et al., 2012). The left IFG was chosen as the region most sensitive to verbal WM tasks while the right cerebellum lobule VI/VIIa/CrusI was included for its specific associations with the articulatory loop (Emch et al., 2019; Marvel and Desmond, 2010). The bilateral supplementary motor area (SMA) and right dorsal premotor cortex (dPMC) were selected as most sensitive to non-verbal WM tasks (Owen et al., 2005; Rottschy et al., 2012). Both left and right coordinates for identified regions were chosen from peak coordinate lists within Rottschy et al. (2012) to account for the reduced left lateralization expected from a younger participant group with a high percentage of DD participants. In cases where two peaks of activation were identified for an anatomical region, both were made into individual ROIs as no anatomical overlap was detected after creating spherical activation maps (see Table 2; Fig. 6).

Differences in ROI activation between subject groups and word conditions were assessed via three-way (group × stimulus × region) ANOVA. Separate analyses were performed between the right (2 × 3 × 9) and left (2 × 3 × 8) hemisphere to account for limitations in power. Post-hoc analyses were used to further assess differences in ROI activity between subject groups for each word condition, via (group × region) ANOVA, and to assess differences within individual ROIs between word conditions via (stimulus × region) ANOVA. To evaluate whether or not differences in activity were associated with a significant change in ROI activation between standard and oddball trials, an additional False Discovery Rate (FDR) corrected analysis by group and stimulus condition was conducted. Significant change in activation was determined by one-sample t-tests across TD and DD groups individually with a test value of 0 (C.I. 95%). Data were bootstrapped to 1000 samples with bias corrected accelerated adjusted confidence intervals and corrected for multiple comparisons across each stimulus condition with an FDR correction.

## 3. Results

### 3.1. Behavioural analysis

Participants performed well on the FastLoc-R/A task, with a total mean accuracy of *M* = 0.88 (*SD* = 0.11) across all trials. Errors of omission during oddball trials (23%, *SD* = *0.19*) were more than twice as common as errors of commission during standard reading trials (9%, *SD* = 0.11), which can be attributed to the increased difficulty of the 1-back task within oddball trials. Notably, there was a significant main effect of subject group on task accuracy across all stimulus types [mean accuracy: *F*(1,147) = 20.54 *p* = <.001]. TD participants had extremely high accuracy in all word conditions (familiar word: *M* = 0.99 *SD* = 0.03, pseudoword: *M* = 0.98 *SD* = 0.03, false font: *M* = 0.97 *SD* = 0.05) in comparison to DD participants (familiar word: *M* = 0.87 *SD* = 0.12, pseudoword: *M* = 0.87 *SD* = 0.13, false font: *M* = 0.86 *SD* = 0.15). Individual t-tests revealed this difference in accuracy was significant across familiar word [*t*(95.94) = −7.21, *p* = < .001] pseudoword [*t*(95.90) = −6.92, *p* = < .001], and false font [*t*(95.60) = −5.60, *p* = < .001] stimuli. In both groups task accuracy was lower in false font trials compared to familiar word and pseudoword trials, but this difference did not reach significance in either group.

Between group comparison of CVLT-C measures are recorded in Table 1. Significant group difference was found in long delay cued recall [*t*(50.90) = −2.29, *p* = .022] with TD participants showing a higher average in total correct scores. Group difference in long delay cued recall might suggest TD participants showed stronger verbal memory than DD participants. However, there was no group difference found for correct recognition hits, which was also a loading variable for the verbal memory factor. Percent recall consistency, which was the main contributor to the executive function factor also showed no significant group differences in performance. Overall these results suggest group differences in verbal WM may lie primarily within verbal memory processes related to long term storage rather than within non-verbal executive control. All CVLT-C measures strongly correlated with measures of performance accuracy.

#### 3.1.1. Brain-behaviour correlations

Correlational analysis of task performance on ROI activity was conducted across all participants, rather than by group, to best capture the normal range of all measures. Performance accuracy was negatively correlated with activity in the right dPMC (*r* = −.234, *p* = .020) in the false font condition, the right IFG (*r* = −0.32, *p* = .001) in the pseudoword condition, and the bilateral SMA (left SMA *r* = −0.33, *p* = <.001; right SMA *r* = 0.34, *p* = <.001) and right rPFC (*r* = −0.34, *p* = <.001) in the familiar word condition. With increased activity in these regions participants saw a *decrease* in task accuracy, suggesting these primarily right hemisphere regions are inefficient when responding to verbal stimuli. All correlations remained significant following additional median split testing to account for ceiling effects in performance accuracy.

There were a number of ROIs which showed significant correlation with CVLT-C measures in the pseudoword condition across all participants (Table 3). Activity change in the left MFG and right rPFC had a positive correlation with long delay cued recall, and recognition hits – both variables associated with the verbal memory factor. Increased activity in the left MFG and right rPFC during oddball trials may be associated with increased verbal recall in young readers. However, this finding is at odds with the negative relationship found between task accuracy and right rPFC activity in unrelated word trials. Increased activity in the left SPL, right IPS, and right crusI of the cerebellum all had a negative correlation with percent recall consistency – the measure best associated with executive functioning. These results suggest increased activity in right and left parietal regions as well as the right cerebellum is *negatively* associated with non-verbal executive functioning skills related to immediate encoding. The false font and unrelated word conditions did not have ROI activity that correlated with any CVLT-C measures associated with the three-factor model.

### 3.2. ROI analysis

There was a significant three-way interaction between subject group, word condition, and ROI with regards to the change in activation during oddball trials in the right hemisphere [*F*(7.40, 718.08) = 2.45, *p* = .015], with a main effect of subject group [*F*(1,97) = 5.76, *p* = .018] and region [*F*(5.93, 575.11) = 2.23, *p* = .040]. Three-way interaction was not observed in the left hemisphere [*F*(10.1, 975.5) = 1.23, *p* = .263], however a two-way interaction between subject group and stimulus was observed [*F*(2,194) = 3.47, *p* = .033], with a main effect of ROI only [*F*(5.71, 553.88) = 3.53, *p* = .002]. As a result of the interactions between subject group and stimulus variables, post-hoc analyses were conducted to assess differences between groups within each stimulus condition in the right and left hemisphere.

#### 3.2.1. Between group comparisons

**Fig. 2.**
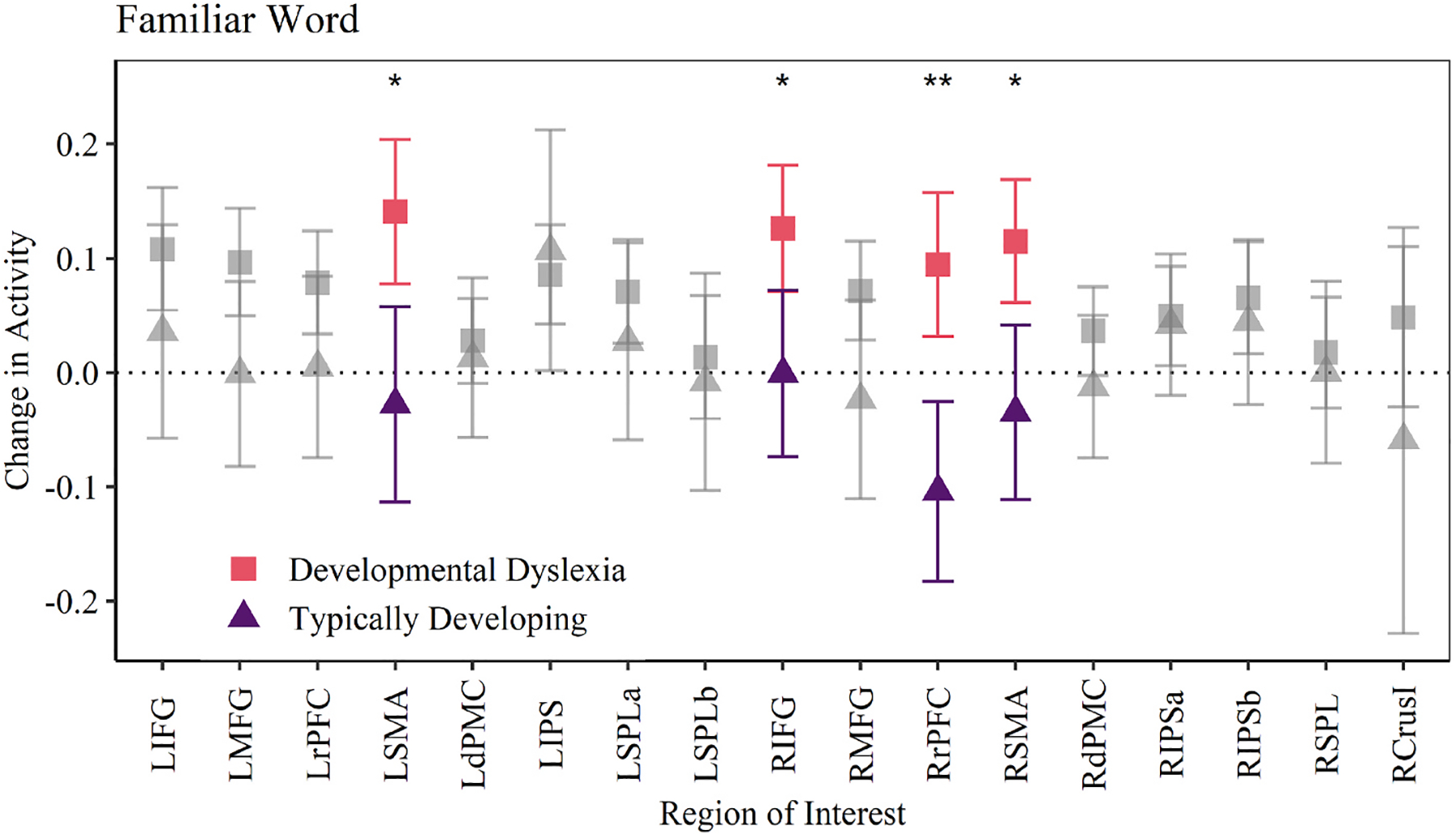
Comparison of mean activity change between groups in familiar word trials. Significant regions include left supplementary motor area, right inferior frontal gyrus, right rostral prefrontal cortex, and right supplementary motor area. **p < .01, *p < .05.

In the familiar word condition, post-hoc ANOVA revealed interaction between subject group and ROI in the right hemisphere [*F*(5.24, 507.80) = 2.381, *p* = .035]; the same interaction was found the left hemisphere as well [*F*(5.55, 538.20) = 2.281, *p* = .039]. Pairwise comparisons evaluated differences of individual ROI activity between groups, corrected by False Discovery Rate (FDR). Comparisons of right and left hemisphere ROI for familiar word stimuli are shown in Fig. 2. Significant differences in activity were identified between TD and DD groups in the inferior frontal gyrus [*t*(49.73) = 2.84, *p* = .025], rostral prefrontal cortex [*t*(53.17) = 4.026, *p* = .002] and supplementary motor area [*t*(46.03) = 3.29, *p* = .013] in the right hemisphere, and in the supplementary motor area [*t*(48.33) = 3.25, *p* = .026] in the left hemisphere. In all cases group differences appear to be driven by a significant increase in activity in response to oddball trials within the DD group. These regions, which include the right IFG, provide an alternative mechanism for maintaining verbal memory in children with DD who may otherwise struggle to quickly recognize familiar words. In comparison, the TD group shows little change in ROI activity in response to oddball trials, suggesting they did not need to recruit the WM network to hold familiar words in memory.

**Fig. 3.**
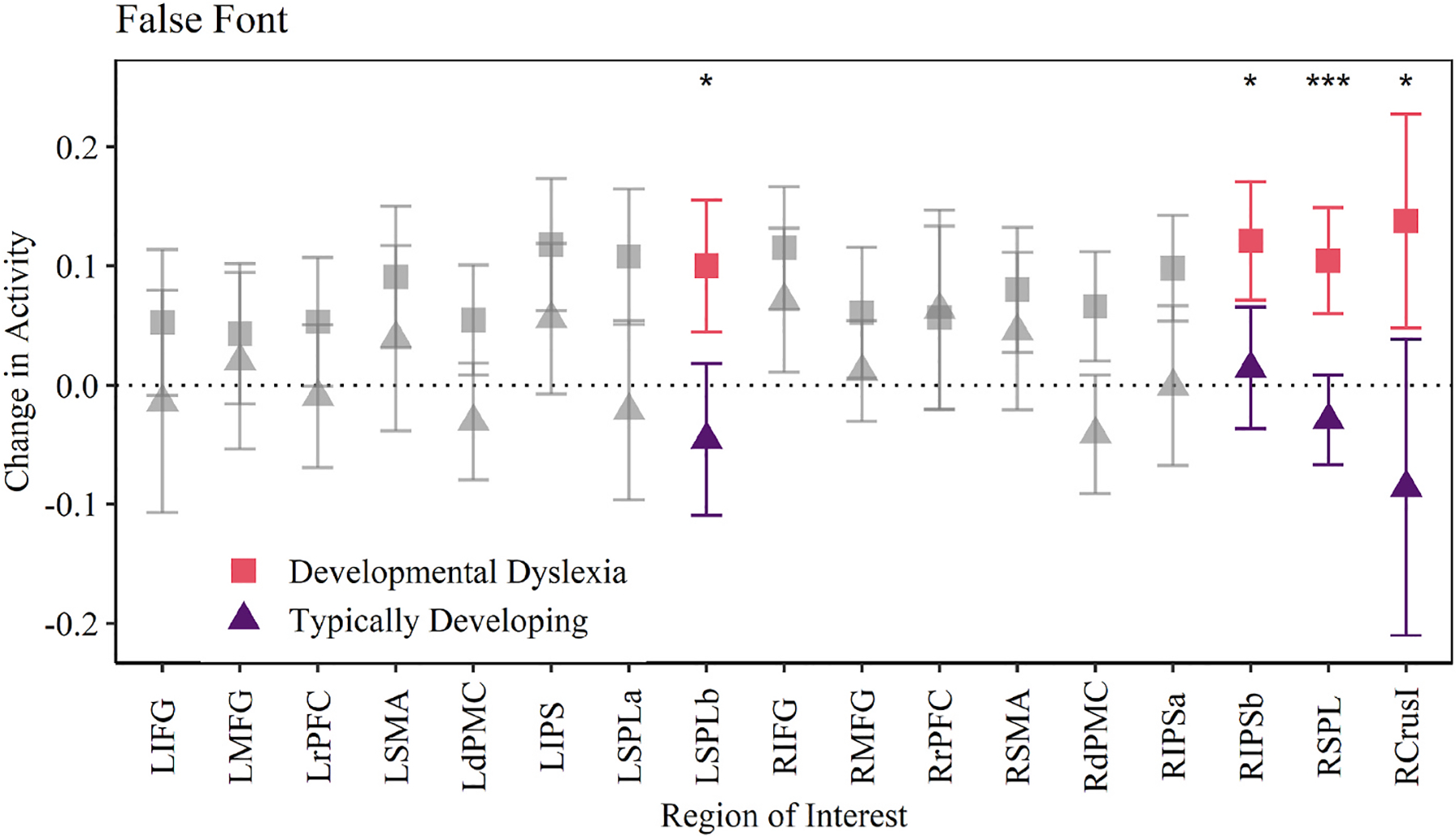
Comparison of mean activity change between groups in false font trials. Significant regions include left superior parietal lobule^b^, right intraparietal sulcus^b^, right superior parietal lobule, and right cerebellum crusI. ***p < .001, *p < .05.

Comparison of subject group and ROI activity in the false font condition found a main effect of subject group in the right hemisphere [*F*(1,97) = 5.01, *p* = .027], and again within the left hemisphere [*F*(1,97) = 4.11, *p* = .045]. FDR corrected pairwise comparisons shown in Fig. 3 indicate the DD group had significantly higher mean activity than controls within the dorsal premotor cortex [*t*(62.47) = 3.23, *p* = .013], intraparietal sulcus [*t*(67.01) = 3.05, *p* = .018], superior parietal lobule [*t*(81.36) = 4.65, *p* < .001], and crusI of the cerebellum [*t*(47.42) = 2.99, 0.020] in the right hemisphere. The superior parietal lobule in the left hemisphere also saw significant group differences in activity [*t*(58.66) = 3.52, *p* = .020]. Group differences in the false font condition echo the results found in the familiar word condition – the DD group responded to oddball trials with an increase in ROI activity while the TD group had little change in activity between standard and oddball trials. In this study false font stimuli were intended to act as a non-verbal control. Our results may suggest DD participants used a non-verbal memory strategy across all conditions. Alternatively, it may indicate false font stimuli are not truly non-verbal.

#### 3.2.2. Within group comparisons

Following between group comparisons, a within group post-hoc analysis was conducted to better assess the effect of stimulus on ROI activity change while holding subject group constant. Looking first at the TD group, two-way ANOVA in the right hemisphere revealed an interaction effect between stimulus and ROI [*F*(6.26,131.36) = 2.87, *p* = .011]. Follow up analyses showed there was a significant main effect of stimulus condition on activity in the right rostral prefrontal cortex [*F*(2,42) = 4.02, *p* = .025], dorsal premotor cortex [*F*(2,42) = 4.42, *p* = .018], and crus I of the cerebellum [*F*(2,42) = 4.64, *p* = .015]. Within the right dPMC and Crus I this effect appears to be driven by increased activity in the pseudoword condition compared to familiar word or false font conditions. TD participants may utilize these regions to support phonetic decoding when stimuli cannot be recognized by word recognition.

Supporting this idea, in the left hemisphere there was a main effect of stimulus condition [*F*(2,42) = 3.99, *p* = .026] and ROI [*F*(4.10, 86.01) = 3.35, *p* = .013], with the left inferior frontal gyrus [*F*(2,42) = 3.30, *p* = .047] and left dorsal premotor cortex [*F*(2,42) = 5.19, *p* = .010] showing a significant effect of stimulus type on activity. Once again these regions show a pattern of increased activity in response to pseudoword stimuli over false font or familiar word stimuli, suggesting TD participants utilize the WM network to support phonetic decoding. Pairwise comparisons were conducted to further evaluate differences between pseudoword stimuli and false font or familiar word stimuli, but no differences remained significant after FDR correction.

**Fig. 4.**
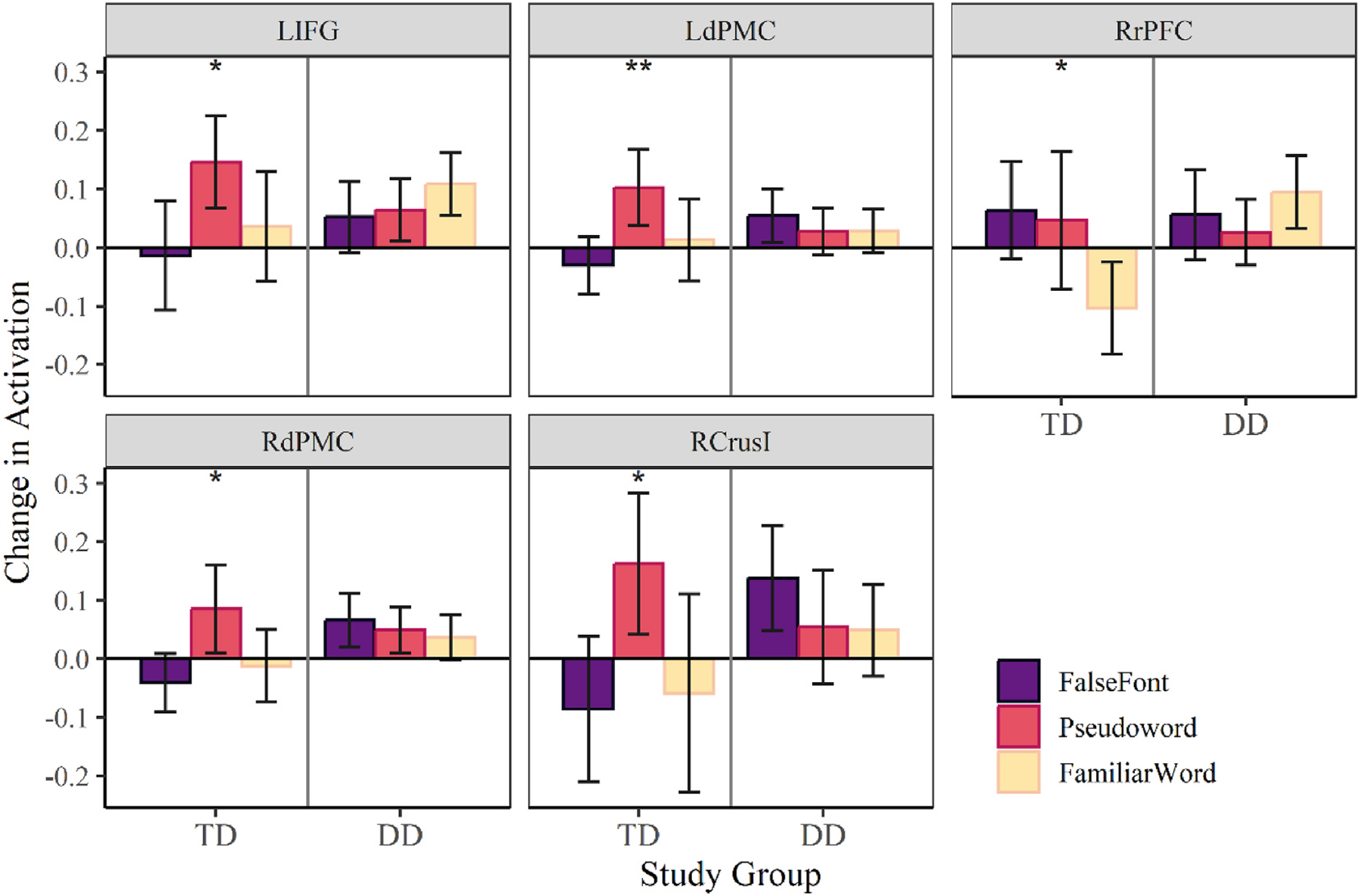
Comparison of mean activity in regions of interest which showed significant differences in activity across stimulus type. Pairwise comparisons did not remain significant after correction, but suggest greatest differences in activity occurred between false font and pseudoword conditions in the left inferior frontal gyrus, left dorsal premotor cortex, right dorsal premotor cortex, and right cerebellum crusI (TD group). Differences in TD group activity between false font and familiar word conditions were also suggested in the right rostral prefrontal cortex. There were no main effects of stimulus type within individual ROIs in the DD group. **p < .01, *p < .05.

The DD group, in comparison, showed no main effect of stimulus in the right hemisphere [*F*(2,152) = 0.92, *p* = .402], but did show an interaction between stimulus and ROI in the left hemisphere [*F*(9.82, 746.24) = 2.01, *p* = .031]. No main effects of stimulus were found within individual left hemisphere ROIs in the DD group, suggesting change in activity due to oddball trials was not driven by use of different memory strategies in response to different stimulus types in this group. These results may indicate that DD participants are more likely to utilize the same memory strategy to respond to oddball trials regardless of stimulus type. Alternatively, DD participants may experience a general deficit in WM requiring increased recruitment in WM regions to correctly respond to oddball trials. Contrasting effect of stimulus type on activity between TD and RD groups is shown in Fig. 4 for ROIs in which a main effect was observed.

#### 3.2.3. Signal-noise ratio

**Fig. 5.**
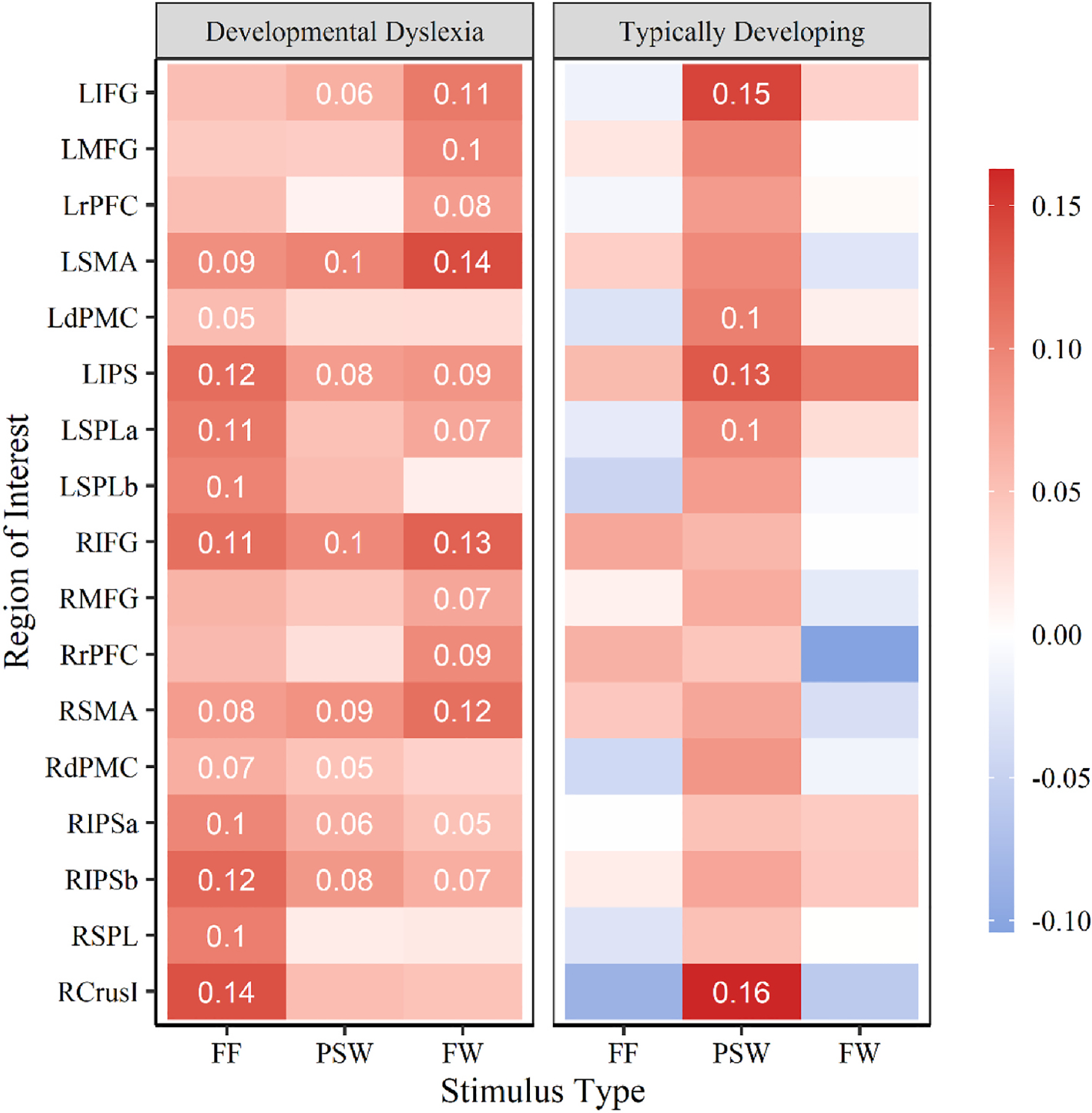
ROI activation change in response to oddball trials over standard trials between groups. Change in activation is shown for each of the following three stimulus types: false font (FF), pseudoword (PSW), and familiar word (FW). Mean activation is displayed for ROIs which showed significant change (p < .05) after FDR correction.

**Fig. 6.**
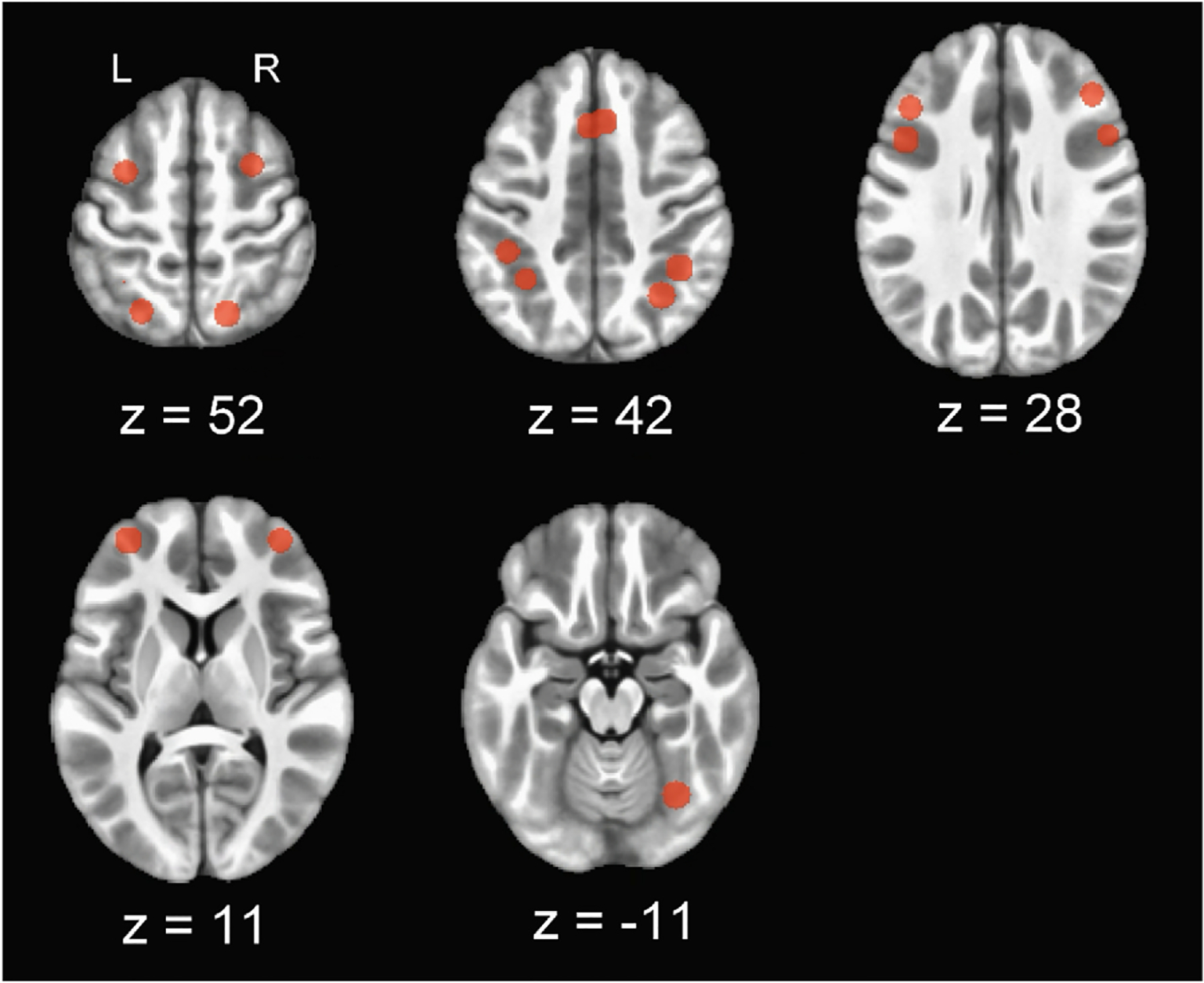
Illustration of *a priori* ROIs in axial plane. Pictured at z = 52, left dPMC, left SPLb, right dPMC, right SPL. At z = 42, left SMA, left IPS, left SPLa, right SMA, right IPSa, right IPSb. At z = 28, left MFG, left IPS, right MFG, right IPS. At z = 11, left rPFC, right rPFC. At z = −11, right crus I. Z = Haskins Pediatric coordinates in lpi orientation.

An additional signal-to-noise analysis was conducted to better conceptualize which ROIs showed change in WM activity that differed between reading and oddball trials (Fig. 5). It is possible for group differences to be found in ROIs that do not have significant change in activity between reading and oddball trials. Such group level differences would be difficult to attribute to WM activity. This additional analysis showed the DD group had a significant increase in activation in response to oddball trials across several right and left hemisphere ROIs in the false font, pseudoword, and familiar word conditions. All of the differences in ROI activity previously identified between groups occurred in regions which saw significant increase in activity within the DD group, and no significant change in activity within the TD group. In the TD group, evidence of significant change in activity in response to oddball trials was only observed in the pseudoword condition, adding further support that TD participants utilized the WM network to support phonetic decoding. Regions which had a significant increase in activity include the left inferior frontal gyrus, dorsal premotor cortex, intraparietal sulcus, and superior parietal lobule, as well as the right crus I of the cerebellum.

## 4. Discussion

The primary aim of this study was to investigate activity in the WM network relative to single word reading skill in children with developmental dyslexia and their typically developing peers. Using the FastLoc-R/A task, we paired single word reading trials with oddball trials that require active assessment of stimuli to identify group differences in WM network recruitment that were specific to the reading process. Familiar word, pseudoword, and false font stimuli were included to assess if group differences persisted across word types which, if observed, might reflect reliance on different word reading strategies between groups. A subtraction analysis identified changes in WM network activity in response to 1-back oddball trials over standard reading trials. To assess the relationship between brain and behaviour, WM network activity was correlated with verbal WM measures in the CVLT-C.

Through this study we identified significant group differences in WM network activity in response to false font and familiar word stimuli, but not pseudoword stimuli. These differences reflect an increased reliance on WM to support reading in the DD group, despite lower task performance. A main effect of stimulus type on ROI activity was specific to the TD group, suggesting WM recruitment was sensitive to pseudoword stimuli, but did not survive pairwise comparisons. ROI activity during pseudoword trials was also found to moderately correlate with cognitive measures of verbal WM in the CVLT-C, suggesting individual differences in WM network activity are associated with verbal memory and phonetic decoding. Overall, these findings suggest a differential role for WM during single word reading in DD and TD groups, and contribute to a growing literature characterizing the specific role of executive functioning in reading development (Peng et al., 2018; Weiss-Croft and Baldeweg, 2015).

### 4.1. Group comparisons

Our findings indicate children with DD recruit the WM network to a greater degree during single word reading than do their typically developing peers. Group differences indicated the DD group showed increased bilateral ROI activity in the familiar word and false font conditions compared to the TD group, and that both groups shared similar increases WM network activity in the pseudoword condition. Despite this, the DD group had significantly lower task performance across *all* stimulus conditions, suggesting a complex relationship between WM network activity and reading performance in struggling readers. Children with DD often utilize more cognitively effortful strategies during word reading (Waldie et al., 2017). A number of studies have shown the deficits in phonological awareness present in DD impede the development of a mental lexicon which would allow readers to effortlessly retrieve familiar words (Berninger et al., 2008; Dehaene--Lambertz et al., 2018; Hoeft et al., 2011). As a result, readers with DD show an increased reliance on a number of WM regions to support verbal memory during reading when compared to age-matched controls (Chyl et al., 2021). These regions include the left IFG, bilateral MFG, bilateral SMA, and the left precentral gyrus (Morken et al., 2017; Richards and Berninger, 2008). In our study, the DD group had consistent increase in WM activity across all trial conditions but saw lower task performance than controls, potentially reflecting a similar compensatory reliance on less efficient WM systems.

This interpretation is supported by our findings in the TD group, in which significant changes in WM activity were found only in the pseudoword condition – as would be expected by a reader accessing a phonological decoding strategy only when necessary (Pugh et al., 2001). In response to pseudoword stimuli, significant change in activity in the TD group was limited to left lateralized ROIs, with the exception of the right crus I of the cerebellum which is thought to play a part in subvocal rehearsal in conjunction with the left IFG (Stoodley et al., 2012; Thomason et al., 2009). The right crus I and crus II in the right cerebellum are prevalent in reading research, with neuroimaging studies showing these regions are structurally connected to the left lateralized reading network in typically developing children (Alvarez and Fiez, 2018) and adults (Pleger and Timmann, 2018). Interestingly, our study revealed a *negative* correlation between the right crus I and CVLT-C measure percent recall consistency. Greater right crus I activation may have benefitted TD participants in accurately responding to the FastLoc-R/A task, but not with recalling target words across learning trials on the CVLT-C. Functional parcellation has shown the cerebellum has strict boundaries between functions (King et al., 2019). Activity in our coordinates representing the right crus I may therefore be best associated with immediate rehearsal or short-term memory maintenance.

#### 4.1.1. False font stimuli

Group level differences were an unexpected finding in the false font condition. False font stimuli were intended to be a non-verbal control condition and were expected to produce a similar response during oddball trials in both DD and TD participants. The fact that we did not find this could be attributed to traits of the false font stimuli which did not agree with our subtraction method used to isolate change in ROI activity. In the TD group, false font stimuli failed to produce a significant change in activity in *any* left or right hemisphere ROIs, despite being stimuli which could not benefit from word recognition on oddball trials. This may be because the false font stimuli were the only non-word stimuli out of six total visual conditions, constituting only 1/6th of trials. As such, they may have produced activity in the WM network due to their novelty during *both* the standard reading and 1-back oddball trials. This is a potential confound that would reduce our ability to identify change in activity across standard reading and oddball trials. Alternatively, false font trials may not have required the same level of attentional resource management as pseudoword stimuli when verifying oddball trials, as individual items in this condition cannot be decoded. It may be pertinent to re-evaluate the reliability of non-verbal control conditions that use Wingdings or similar symbol-based fonts.

It may also be possible that there are true group differences in the way non-verbal stimuli are processed in the WM network, which would invalidate false font stimuli as a control measure. Increased visuospatial WM skill in DD populations has been described as a compensatory mechanism for verbal deficits and may indicate use of a visual WM strategy to complete oddball trials instead (Chen et al., 2016). If the DD group was relying primarily on visual information across all trials, it may partially explain the group differences in the false font condition and the similar ROI activity DD participants had across all three stimulus types. Previous research has shown that children with DD are more likely to use a sequential visual memory strategy over a verbal one, although verbal memory is better suited to sequential recall (Frijters et al., 2011; Smirni et al., 2020). It is worth noting that there is a general belief of increased visuospatial WM in DD participants compared to controls (D’Mello and Gabrieli, 2018), which was not found in our study via WASI-2 measures or by direct comparison of task performance in the false font condition. Nevertheless, it is possible DD participants were using a sequential visual strategy for the FastLoc-R/A task, even if it was less successful – resulting in lower task accuracy compared to controls.

### 4.2. WM activity across stimulus types

The DD group had no main effects that would indicate stimulus type influenced ROI activity. Additionally, our signal-to-noise analysis showed six ROIs in this group (right IFG, SMA, IPSa, IPSb; left SMA, IPS) had a persistent increase in activation in response to stimulus conditions. Bilateral activity in the SMA and IPS has been previously associated with response inhibition during both verbal and spatial tasks (Nee et al., 2013; Osada et al., 2019). Likewise, activity in the right IFG has been associated with attentional shifting and increased salience of attention to verbal, visual, and spatial cues (Hampshire et al., 2010). Response inhibition and shifting are functions representing attentional control rather than memory storage and may suggest DD participants relied more on the central executive when responding to oddball trials (Arrington et al., 2019; Camos and Barrouillet, 2014). Response inhibition may help regulate withholding response to standard trials and activating response on oddball if DD participants struggled to quickly identify stimuli. Another theory suggests that the SMA in conjunction with the superior frontal sulcus and bilateral IPS may play a part in the updating role of WM, which shifts attentional allocation based on priority (Nee et al., 2013).

Although DD has been previously defined as a presence of reading difficulties despite normative general cognitive skills, it is now acknowledged that difficulties in executive functions and motor-coordination can also occur in the population (Snowling and Hulme, 2012). Previous studies have reported finding reduced phonological loop capacity (Fischbach et al., 2014; Follmer, 2018) and central executive memory span (Georgiou et al., 2008) in DD populations. These findings may further explain the negative correlation found between task performance and activity in the right IFG, right rPFC, and bilateral SMA. Each of these regions had increased activity in the DD group in our study. Irrespective of origin, persistent ROI activation across verbal and non-verbal stimuli indicates DD participants may be utilizing a less efficient language-independent strategy when responding to oddball trials. Conversely, it may point towards a general deficit in memory span which necessitates support from the central executive. Deficits in the active maintenance of WM has been identified using both verbal and visuo-spatial modalities in children with DD (Beneventi et al., 2010; Fischbach et al., 2014).

#### 4.2.1. Suggestions of stimulus sensitivity in typical readers

Analysis of active ROIs across stimulus type revealed the TD group had a main effect of stimulus condition in a number of ROIs, which was not present in the DD group. This effect suggests activity in these regions may be driven by individual differences in stimuli, and particularly between pseudoword and false font trials. The left IFG and right crus I of the cerebellum have strong functional connections to dorsal and ventral reading regions and have been associated with verbal working memory (Alvarez and Fiez, 2018; Emch et al., 2019; Stoodley and Schmahmann, 2009). With this in mind, it is evident why these regions might have increased activation in response to pseudowords. Although a main effect of stimulus type was identified in the right Crus I, left IFG, and bilateral dPMC in our study, this effect did not show up as significant after correction for pairwise comparisons in our larger model. While our findings did not reach significance, the main effects are in line with previous literature suggesting TD children at this age have an increased salience in the WM network to novel word information and prioritize phonological decoding strategies for unfamiliar words (Facoetti et al., 2010; Schlaggar and Church, 2009).

### 4.3. Brain-behaviour correlations

Individual differences between CVLT-C measures and ROI activity appear to be faceted by CVLT-C factor loadings of verbal memory and executive functioning. Long delay cued recall and correct recognition hits were associated with the verbal memory factor and shared a positive relationship with ROI activity in the left MFG and right rPFC. Similarly, recall consistency, which acted as the primary measure related to executive functioning, shared a negative correlation with ROI activity in the right IPS, left SPL, and right CrusI. No other correlations were found between ROI activity and factor-loading CVLT-C measures in any stimulus condition. A cautious interpretation of these results suggests individuals with stronger verbal memory stores are able to utilize prefrontal regions to aid immediate recall, while those with poorer maintenance of verbal information – possibly related to the central executive – activate parietal ROIs and the cerebellum to a greater degree when trying to recall information. There may be some support for this interpretation. Increased activity in the left prefrontal cortex is associated with reading improvement in children with dyslexia (Partanen et al., 2019), but this activity might be at its most beneficial when scaffolding communication between reading regions rather than compensating directly (Aboud et al., 2018). Additional research suggests reduced resting-state connectivity between the left MFG and left IPS is a characteristic of dyslexia and inattention (Zhou et al., 2015), with the lowest connectivity found among children who do not respond well to intervention (Koyama et al., 2013). Increased activity within the WM network at the cost of executive performance may be a reflection of a central executive that is unable to meet the demands of a task while maintaining the memory systems (Lavie, 2010; Morey and Cowan, 2005). With that said, none of these regions further correlated with task accuracy in our study.

Struggling readers rely more heavily on bilateral frontal and right temporo-parietal regions to compensate for disrupted connectivity within the posterior reading network (Hoeft et al., 2011; Pugh et al., 2001). Although this is often reported, the effect of these differences on reading outcomes varies considerably across studies and can be difficult to parse. In our study, increased WM network activity was a prevalent finding in the DD group; however, there were only a handful of ROIs which negatively correlated to task performance across both groups. Of these regions, individual differences can only be suggested in the right IFG, which was negatively correlated with task performance in the pseudoword condition. Pre-intervention activity in the right IFG has been associated with increased response to reading intervention (Barquero et al., 2014; Chyl et al., 2021; Hoeft et al., 2011). However increased right IFG connectivity to reading regions is also characteristic of dyslexia (D’Mello and Gabrieli, 2018; Finn et al., 2014) and is associated with poorer development of word recognition skills (Morken et al., 2017). Increased activity in the right IFG and right parietal regions may therefore reflect engagement of compensatory reading strategies, as previous studies have suggested (Chyl et al., 2021; Partanen et al., 2019; Waldie et al., 2017).

It is important to note that intervention studies measure the association of the right IFG on reading gains, and not immediate skill or task performance. Thus, our negative association between the right IFG and task performance, and additionally between parietal ROIs and recall consistency in the CVLT-C, may not be as counterintuitive as they first appear. Research by Aboud et al. (2018) indicates that executive functioning in the PFC may contribute positively to reading gains when mediating increased connectivity of the reading network, but not when directly compensating for reading regions. If this is the case, increased right hemisphere activity in children with DD may represent compensatory activity that varies in its efficiency, resulting in an unclear relationship between WM activity and early reading development (Koyama et al., 2013; Partanen et al., 2019). Future research may gain more insight by considering connectivity between WM and reading regions during early reading development, instead of activity alone. Based on the findings from our study, we propose the right IFG, right IPS, and right rPFC may be regions of particular interest, due to the negative brain-behaviour correlations and group differences found in these regions.

### 4.4. Limitations

There are a number of limitations in the current study which should be considered. Participants within the DD group who met criteria for attention-deficit/hyperactivity disorder (ADHD; n = 31) and/or specific language impairment (DLD n = 19) were not excluded from the study to better reflect their ubiquitous co-occurrence in DD populations. ADHD co-occurs with DD at a rate far above chance, sharing deficits in cognitive processing speed that are attributable to common genetic factors (Li et al., 2020; Willcutt et al., 2007). Similarly, DLD and DD share common deficits in cognitive skills such as phonological processing and verbal short-term memory (Pennington and Bishop, 2009; Spanoudis et al., 2019). Choosing to include participants with comorbid developmental disorders may limit the capacity of this study to attribute WM activity to specific reading behaviours. However, by including these participants, the current study provides results from more typical DD children with a spectrum of co-occurring cognitive and language-based strengths and weaknesses. Consequently, we would argue that this does not greatly diminish the goal of this study to identify functional differences in WM network activity between struggling readers and controls.

In addition, the FastLoc-R/A task required participants to press a button in response to oddball trials. There is potential our study may have identified some changes related to such motor activity rather than just WM activity. The bilateral SMA and dPMC are two motor regions which saw significant activation change in this study. However, it should be noted that significant activity in these regions was not found consistently between the two groups, despite both having a performance accuracy above 85%. Another area of concern was the false font condition, which was intended to act as a non-verbal control but produced some unexpected results. This may be due to the complexity of the Wingdings font in comparison to words or single shapes/images, or due to the previously mentioned oddball effect given that this condition was uncommon among all other word conditions. Future research utilizing similar tasks may consider changing the non-word condition to letter strings to reduce potential confounds.

## 5. Conclusion

The findings of this study show different patterns of WM network between TD and DD children when responding to a single word decoding task. Pseudoword stimuli elicited increased WM activity across both groups, as would be expected from a phonological decoding strategy in TD participants. However, the DD group additionally showed increased WM activity in response to familiar word and false font stimuli which was significantly greater than their typically developing peers. This pattern of network recruitment suggests DD participants utilized WM in response to all stimuli, including false font, which may reflect use of a single non-verbal strategy for all trials. Conversely, WM recruitment may also reflect increased reliance on attentional control to coordinate a correct response to standard and oddball trials. When responding to oddball trials, DD performance was significantly less accurate than TD performance, suggesting increased WM network activity in DD participants is not fully compensating for poorer reading skill. Brain-behaviour correlations revealed negative association between ROI activity and task performance, and between right parietal regions and the right cerebellum and behavioural measures of executive functioning. Together these results suggest that WM activity has a markedly different contribution to the development of early reading skills in struggling readers than it does in typically developing readers. Future research will be necessary to begin to characterize the effects of individual differences in WM network activity on early reading skills and reading outcomes.

## Figures and Tables

**Table 1:** Descriptive statistics between groups.

Variable	Dyslexia (n = 77)	Controls (n = 22)	Comparison
	*M (SD)*	*M (SD)*	*t (p)*
Age	9.3 (0.69)	9.25 (0.57)	
ADHD Diagnosis (%)	40.26	0	
DLD Diagnosis (%)	23.38	0	
WASI2 - Matrix Reasoning	12.82 (4.15)	16.05 (4.28)	−3.19 (.002)
NEPSY - Auditory Attention	27.25 (2.9)	28.5 (2.04)	−1.90 (.061)
*Reading Composites*			
WJ3 Broad Score	81.91 (8.97)	111.73 (6.38)	−14.56 (<.001)
WJ3 Basic Score	86.71 (8.36)	111.23 (6.78)	−12.60 (<.001)
TOWRE-2	72.39 (8.93)	105.5 (11.05)	−14.52 (<.001)
SRI2 Reading Quotient	77.09 (10.56)	107.77 (15.15)	−10.84 (<.001)
*CVLT Measures*			
Long Delay Cued Recall	8.76 (2.64)	9.91 (1.77)	−2.29 (.022)
Correct Recognition Hits	13.1 (1.79)	13.73 (1.49)	−1.50 (.136)
Percent Recall Consistency	75.62 (14.03)	78.62 (10.59)	−0.93 (.356)

WASI2, NEPSY total correct score. WJ3 Broad, WJ3 Basic, TOWRE-2, SRI2 standardized score (≤85 = 1 SD below normal).

CVLT Long Delay Cued Recall, Correct Recognition Hits total correct score.

**Table 2:** Regions of interest in the working memory network.

Region		Cytoarchitectonic location	Haskins Pediatric coordinates		
			X	Y	Z
L. inferior frontal gyrus	IFG	BA 45	−42	12	27
L. middle frontal gyrus	MFG	BA 46	−40	26	25
L. rostral prefrontal cortex	rPFC	BA 10	−32	50	12
L. supplementary motor area	SMA		−2	19	41
L. dorsal premotor cortex	dPMC		−29	0	49
L. intraparietal sulcus	IPS	BA 40/h1P3	−38	−36	45
L. superior parietal lobule^a^	SPLa	BA 7/7 PC	−30	−48	46
L. superior parietal lobule^b^	SPLb	BA 7/7A	−22	−61	49
R. inferior frontal gyrus	IFG	BA 45	46	14	24
R. middle frontal gyrus	MFG	BA 9	39	32	31
R. rostral prefrontal cortex	rPFC	BA 10	34	50	8
R. supplementary motor area	SMA		4	21	41
R. dorsal premotor cortex	dPMC		26	3	49
R. intraparietal sulcus^a^	IPSa	h1P2	37	−43	41
R. intraparietal sulcus^b^	IPSb	h1P3	29	−55	42
R. superior parietal lobule	SPL	7P	15	−62	50
R. cerebellum	CrusI	lobule VI/VIIa Crus I	28	−62	−11

a, b regions with multiple peaks of activation listed under the same anatomical region.

Coordinates in LPI orientation.

Coordinates (MNI) retrieved from Rottschy et al. (2012).

**Table 3:** Correlations between factor related CVLT-C measures and pseudoword ROI activity.

	R. IPSa	R. IPSb	L. SPLb	R. CrusI	L. MFG	R. rPFC
Long Delay Cued Recall	−.100	.075	.175	.022	.213^a^	.242^a^
Correct Recognition Hits	−.035	.034	−.011	.089	.269^b^	.285^b^
Percent Recall consistency	−0.278^b^	−.352^b^	−.221^a^	−.290^b^	−.169	−.050

a Correlation significant at .05 level (2-tailed).

b Correlation significant at .01 level (2-tailed).

## Data Availability

Data will be made available on request.
